# Vaccination against infectious agents and allergen-specific immunotherapy: A critical analysis 

**DOI:** 10.5414/ALX02390E

**Published:** 2023-03-31

**Authors:** Margitta Worm, Oliver Pfaar

**Affiliations:** 1 Division of Allergy and Immunology, Department of Dermatology, Venerology and Allergology, Campus Charité Mitte, Universitätsmedizin Berlin, and; 2 Section of Rhinology and Allergy, Department of Otorhinolaryngology, Head and Neck Surgery, University Hospital Marburg, Philipps-Universität Marburg, Marburg, Germany

**Keywords:** allergen immunotherapy, vaccination, SARS-CoV-2, time intervals, interactions

## Abstract

Allergen immunotherapy (AIT) and vaccination against infectious agents (VIA) are treatments actively interfering with the immune system. This raises the question of whether these therapies influence each other positively and/or negatively if applied simultaneously. For AIT, it should be taken into account that the mechanisms of subcutaneous and sublingual allergen application are in principle similar, but must be assessed in respect to vaccination differently due to their different routes of allergen administration. Here, the immunological mechanisms of both AIT application forms in respect to VIA are discussed in more detail followed by a critical discussion based on the literature and considering current practice.

## Introduction 

Allergen immunotherapy (AIT) modulates the Th2-dependent IgE response leading to reduced or ideally no symptoms to the allergen of interest in a given patient. To date, both subcutaneous and sublingual routes of allergen application have been established [1, 2]. The immunological mechanisms are complex and nowadays very well, but still not fully, understood. Essentially, a change in the immune response occurs at the T-cell and B-cell level with the formation of probably protective T-cell and B-cell responses [3]. 

Vaccination against certain viruses, as well as bacteria, is one of the major medical advance of the 20^th^ century. Even most recently, during the COVID-19 pandemic, the enormous importance of vaccination against viruses in a pandemic situation has been demonstrated. Vaccination, and likewise AIT, induces an antigen-specific acquired immune response with the aim to generate an immune protection against the according antigen. The induction of specific T- and B-cell immune responses are key events upon vaccination against infectious agents, with the development of cytotoxic T- and/or humoral B-cell-dependent immune responses leading to the formation of antigen specific IgA and/or IgG antibodies. 

In the following article, the immunological mechanisms of both forms of therapy (AIT and vaccination against infectious agents (VIA)) will be discussed. Based on the available literature, we critically assess whether or which time intervals should be considered for AIT and VIA if given in time proximity, taking into account prevailing practice. 

## Immune mechanisms in AIT 

The mechanisms of allergen-specific immunotherapy have been increasingly well understood in recent decades. Different cells involved in an allergic immune response are affected by the therapy. Initially, after the application of high allergen doses by either subcutaneous injection or sublingual application, tolerogenic dendritic cells are formed [3, 4, 5]. Subsequently, over several weeks, the appearance of IL-10-expressing T-regulatory cells has been described, which in addition express TGF-β and IL-35 as well as Fox p3 [6, 7, 8, 9, 10]. During the course of allergen-specific immune therapy, an enhanced IgA, but also IgG1 and IgG4 immune response through allergen-specific B cells, both systemically and locally, occurs [11]. These allergen-specific IgA and IgG antibodies compete with allergen-specific IgE and prevent the formation of IgE-allergen complexes. Furthermore, there is an interference with IgE-mediated signaling pathways via Fcε-R1 and Fcε-R2 receptors, which in turn have an impact on the Th2 immune response. In the context of AIT, it should be taken into account that especially the subcutaneous routes may induce systemic immunological effects during the induction phase, which in principle could theoretically interfere in case of a concurrent vaccination, e.g., with virus vaccines. In principle, AIT for inhalant allergens is performed for 3 years, for venom allergy, depending on the severity of the reaction in a given patient, for 3 – 5 years, and in the presence of a mastocytosis even for a lifetime [12]. Therefore, to know whether and to what extent AIT may interfere with a concurrent vaccination is of major importance. Although allergen-specific immunotherapy and vaccination against pathogens have both been performed in patients for more than 100 years [2], there are only very limited or even no data available whether their simultaneous application can somehow interfere. 

## Antigen-specific vaccination against pathogens 

Vaccination is one of the major achievements in medicine in the 20^th^ century. It has recently again demonstrated its enormous medical importance for the general population in the context of the COVID-19 pandemic. Various vaccines against different pathogens are available today. These can be given to individuals as inactivated vaccines, live vaccines, or accentuated live vaccines. In Germany, the Commission for Vaccination provides recommendations regarding the age and kind of vaccination according to current scientific data weighting the risks and benefits to the population (https://www.rki.de/DE/Content/Kommissionen/STIKO/stiko_node.html). 

Most vaccinations take place in early infancy, and since AIT is usually not performed before the age of 5 years, the question about a possible immunological interference of AIT and vaccination against pathogens is usually not given for this age group. The situation is completely different from school age onwards, where booster vaccinations (e.g., tetanus) or vaccinations against HPV, hepatitis B, and others are used depending on the private and/or professional exposure. In principle, the vaccination with pathogens or their components, as in allergen-specific immunotherapy, leads to the activation of various cells, including dendritic as well as T and B cells, with the consecutive development of an antigen-specific humoral immune response, which is intended to protect the organism in the event of a renewed contact with the corresponding pathogen. Basically, vaccinations are divided into so-called basic immunizations and booster vaccinations. Examples of basic immunizations are, for example, those with tetanus and diphtheria toxoid as well as hepatitis B. In these, a longer antigen-specific humoral immune response is formed by the repeated application of the according vaccine (usually 3 times). In order to maintain or strengthen the humoral protection in the long term, the so-called booster vaccinations are applied. Another frequently recurring vaccination is the annually repeated influenza vaccination, which, however, is recommended only for defined population groups e.g., elderly individuals. 

Currently, the COVID-19 vaccine or its booster continues to be recommended primarily for vulnerable groups in Germany. While there was no extensive scientific data on the possible interaction of vaccination with pathogens or their components and AIT before the COVID-19 pandemic, there is currently one paper that addresses whether and to what extent interactions between allergen-specific immunotherapy and vaccinations can occur [12]. Specific immune responses resulting from vaccination depend on the route, dosage, type of vaccine, and adjuvant used. Nowadays, a large variety of different vaccines are available. The most common vaccines most recently used in the context of the COVID-19 pandemic were mRNA-based vaccines. These were first used in a large scale in 2020 and showed significant humoral IgG immune responses in vaccinated individuals 7 – 21 days after the initial and repeat doses, respectively. Due to the global and pandemic nature of SARS-CoV-2 infection, multiple clinical trials have been conducted, providing for the first time a deeper insight into the immunologic consequences of vaccines in a short period of time in numerous patients from diverse cohorts worldwide. In essence, the data show, after SARS-CoV-2 vaccination, the induction of CD4 and CD8 T-cell responses with corresponding cytokine profiles, particularly an interferon-γ response. A Th2 response was not observed [13]. 

## Interference of allergen- and pathogen-based vaccination 

**Figure 1:**
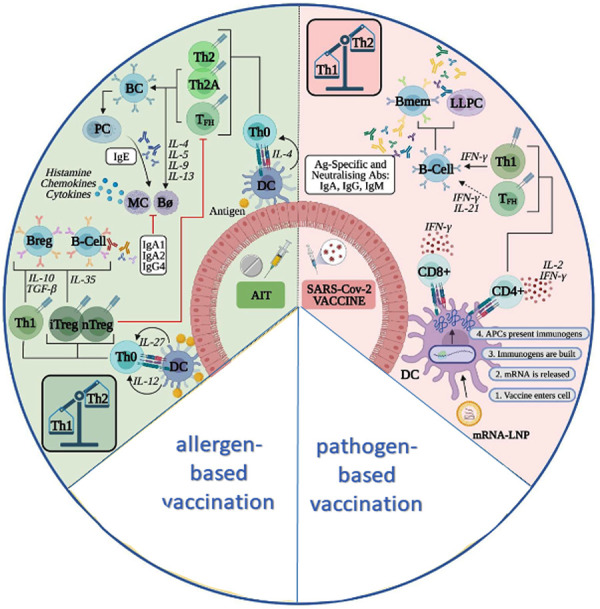
Immune mechanisms during AIT and vaccination against pathogens, modified from Jutel et al. [19].

The guidelines from the allergy societies but also the technical information from various allergen extract manufacturers for preparations for allergen-specific immunotherapy recommend that the application of allergen-specific immunotherapy and a vaccine against pathogens or their components should be given separately by at least a 7 – 14 day interval [14, 15, 16]. However, this recommendation is a pragmatic approach and not based on evidence from controlled clinical trials. A previous retrospective study in 875 individuals showed that systemic reactions were no more frequent in patients who received AIT and vaccination on the same day than when allergen-specific immunotherapy was given alone [17]. The data of vaccination in the context of AIT suggest that booster vaccines in particular can be administered effectively and efficiently in patients on AIT [18]. Also, from a mechanistic perspective, there is no approach that suggests that AIT and vaccination cannot be given simultaneously. It is even possible that there could be beneficial actions from the effects of the vaccination on the innate immune response (Figure 1) [19]. 

Regardless of possible interactions in terms of efficacy of the AIT and pathogen vaccination, it is important in clinical care if in case of the occurrence of an adverse effect when both treatments were given simultaneously a clear assignment of the trigger responsible for the reaction may be impossible. This supports the recommendation of a time interval between these therapies. Therefore, recommendations for COVID-19 vaccination suggested a 7-day interval between AIT and a vaccination for subcutaneous immunotherapy (SCIT) and a 3-day interval for sublingual immunotherapy [16]. Especially during scarce vaccine availability and restrictions due to scheduling, the time intervals of AIT were adjusted according to vaccine availability. Currently, this has changed due to the improved availability of pathogen-based vaccines. As a general rule, vaccination appointments and AIT should be performed in a timed sequence. 

**Figure 2:**
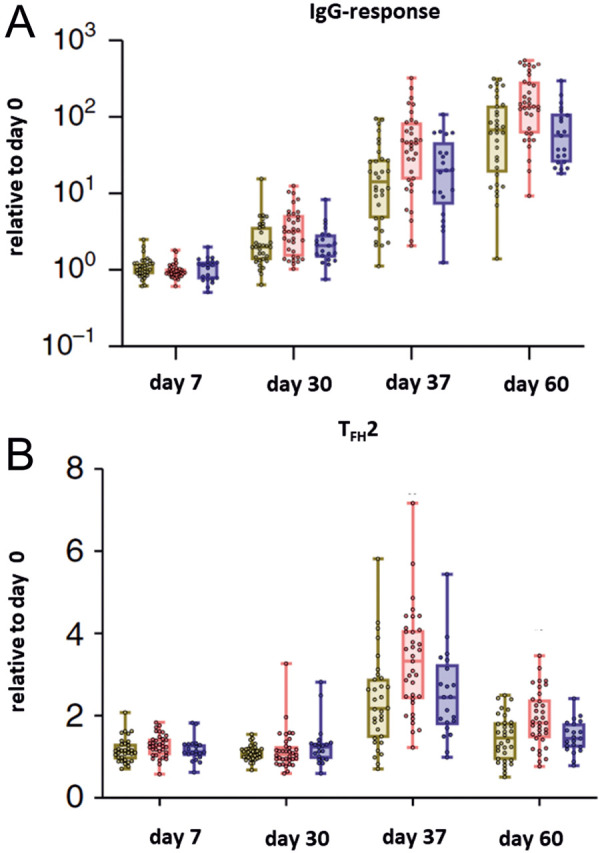
A: Antibody responses to SARS-CoV-2 vaccine (WIBP-CorV, Sinopharm) in patients with allergic rhinitis receiving an allergen-specific immunotherapy, modified from Yao et al. [12]. B: TFH2 response to SARS-CoV-2 vaccine in patients with allergic rhinitis receiving allergen immunotherapy, modified from Yao et al. [12].

Interestingly, in the context of SARS-CoV-2 vaccination, it has been reported that patients with allergic rhinitis develop increased T2 follicular helper cell (TFH-2) responses [20]. In another recently published paper [12], the disease-specific effect on TFH-2 cells was shown to be altered by AIT in patients with allergic rhinitis and AIT compared to untreated patients with allergic rhinitis and controls, in terms of an immune response equivalent to healthy controls. In all groups, an acceptable humoral immune response was seen after vaccination (Figure 2). However, it should be kept in mind that limited patient numbers were studied and further future observations are needed. 

## Conclusion 

Both AIT and vaccination against pathogens modulate the systemic T- and B-cell-dependent acquired immune response. Both treatments are antigen-specific therapies; therefore, direct interactions are not expected, but so-called bystander effects may occur. This refers, for example, to effects of cytokines that are specifically released during an AIT and that may influence other cells of the immune system or the effector cells of the allergic immune response. 

So far, there are very few data on possible interactions of AIT and VIA; however, national but also European and other international guidelines recommend not to apply AIT and pathogen-based vaccination at the same time, but to do so in a time-delayed manner with an appropriate therapy interval. Since both therapies usually do not have to be acutely implemented directly, an appropriate management with the patient is usually uncomplicated and very easy to plan. From a medical point of view, the main reason for the time intervals is the better ability to assess a causal relationship in the event of the occurrence of an adverse reaction. 

## Funding 

None. 

## Conflict of interest 

None.
